# Repeated infusion of killed *Staphylococcus aureus* has increasingly negative effects on milk yield and composition

**DOI:** 10.3168/jdsc.2024-0718

**Published:** 2025-03-12

**Authors:** B.D. Enger, G. Hastings, C.S. Gammariello, M.X.S. Oliveira, K.M. Enger

**Affiliations:** Department of Animal Sciences, Ohio Agricultural Research and Development Center, The Ohio State University, Wooster, OH 44691

## Abstract

Mastitis reduces milk yield of affected mammary glands, but the mechanisms that reduce mammary gland productivity are unclear. We previously aimed to develop a reduced milk yield mastitis model via a single sterile intramammary infusion of oyster glycogen but failed to observe milk yield response even when milk SCC spiked greater than 3,000,000 cells/mL. The aim of this study was to assess the impact of repeatedly challenging mammary glands in a split-udder model to determine if duration of immune response would affect milk yields. Four mid-lactation primiparous cows were used. Quarters of a randomly selected udder half were infused with saline (SAL), whereas the quarters of the opposite udder half were infused with 2 billion cfu of formalin-fixed *Staphylococcus aureus* (FX-STAPH). Intramammary infusions were repeated every 3 d for 12 d; milk yields and composition were measured at each milking (2×/d). Milk yield, composition, and somatic cell scores were similar between udder half treatments before challenge. After the first challenge, cows only displayed subclinical mastitis, and after repeated challenges, bouts of mild clinical mastitis resulted. Somatic cell scores of FX-STAPH udder halves were greater than SAL udder halves at all postchallenge milkings. Milk fat content was unaffected by udder half treatment. Conversely, FX-STAPH udder halves had greater overall mean protein content and lesser lactose content than SAL udder halves. Milk yield of FX-STAPH udder halves were lower than that of SAL udder halves, and the magnitude of depression increased with study duration. Similarly, milk component treatment differences became more pronounced after repeated challenges. The results of this study indicate that the duration of somatic cell count elevation during subclinical or mild clinical mastitis affects the degree of milk compositional change and yield loss.

## Highlights

• First study to repeatedly infuse mammary glands with a sterile irritant intended to cause subclinical or mild clinical mastitis.
• Repeated STAPH infusion had a progressively greater negative effect on milk yield and composition.
• Milk yields of saline-infused udder halves were not meaningfully affected during the study.

## Graphical Abstract

**Figure:**
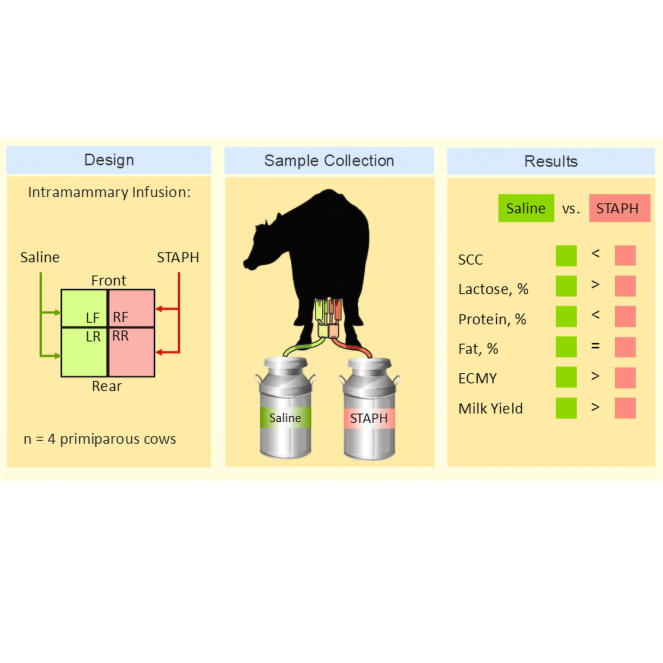
**Summary:** Four mid-lactation primiparous Holstein cows were repeatedly infused with formalin-fixed Staphylococcus aureus (STAPH) and saline in randomly selected udder halves every 3 days over 12 days. Udder half milk composition and milk yields were measured at each milking. Somatic cell counts (SCC) were greater in STAPH than saline udder halves (LF = left front; LR = left rear; RF = right front; RR = right rear). Degree of milk yield loss and compositional change resulting from STAPH challenge became progressively greater after repeated challenges. The results indicate that the duration of SCC elevation is a key factor that determines milk yield losses during subclinical and mild clinical mastitis.

Mastitis is a common disease in the dairy industry that is expensive because of its negative impacts on the quality and quantity of milk synthesized by the mammary gland. Although the greatest monetary loss resulting from mastitis arises from reduced milk production (Huijps et al., 2009), the mechanism(s) of how milk production is reduced is incompletely understood.

Elucidating how mastitis reduces mammary gland productivity is dependent upon controlled intervention studies because associative analyses cannot delineate a cause-and-effect response. For instance, post hoc analyses describe a strong negative relationship between milk SCC and milk production (Jones et al., 1984; Hand et al., 2012), suggesting the degree of immune cell recruitment dictates milk yield (**MY**) losses. However, we recently failed to observe MY losses when milk SCC markedly increased in response to sterile mastitis challenges (Enger et al., 2023; Gammariello et al., 2024). Our previous attempt to develop a split-udder mastitis challenge model did not induce MY responses even though SCC in challenged udder halves exceeded 3,000,000 cells/mL for 8 to 16 h (Enger et al., 2023). Our other attempts that used a single, one-time challenge of different sterile challenge agents of varying dosages were also unsuccessful (B. D. Enger and C. S. Gammariello, unpublished data). We suspected that a single bolus challenge of a mild sterile irritant was insufficient to reduce MY, and that the duration of host response is a significant determinant driving MY loss. The purpose of this study was to test the hypothesis that repeatedly challenging udder halves with killed *Staphylococcus aureus* would reduce MY relative to saline-infused udder halves within the same cow.

Clinically healthy, mid-lactation primiparous Holstein cows (n = 4) were selected from The Ohio State University Krauss Dairy (Wooster, OH) and used in a trial during June 2023. No power calculation was performed. Cows were selected based on previous SCC and MY. Mean daily MY, DIM, and SCC of cows at enrollment was 39.9 kg (SD = 3.3 kg), 70 d (SD = 6 d), and 59,000 cells/mL (range = 13,000–194,000 cells/mL, SD = 78,000 cells/mL), respectively. Cows that were acclimated to tiestalls for at least 4 d were moved to new tiestalls and fed 2×/d. Cows were milked in place 2×/d using procedures previously detailed (Enger et al., 2023) to separate milk from the right and left udder halves to quantify milk weights and determine milk composition. Udder half MY were recorded at each milking and did not differ within each cow by more than 0.7 kg during the 2 d (i.e., 4 milkings) assessed immediately before study commencement.

Beginning at the start of the trial on d 0, immediately after the first daily milking, both quarters of 1 udder half of each cow were each infused with 2 billion cfu of formalin-fixed *Staph. aureus* (**FX-STAPH**), and the contralateral quarters were infused with sterile physiological buffered saline (**SAL**). Treatment allocations were random and were repeatedly administered every 3 d for a total of 5 challenges (i.e., on d 0, 3, 6, 9, and 12). Udder half MY and milk samples were obtained after complete milking from the respective udder half milk buckets immediately before the first challenge and at all subsequent milkings. Milk SCC and components were measured by a commercial DHIA laboratory via a Bentley Fourier Transform Spectrometer and Flow Cytometer (Bentley Instruments, Chaska, MN). Quarters were fore-stripped and examined to identify signs of clinical mastitis at each milking; rectal temperatures were obtained if clinical signs of mastitis were present at the individual milking. Because individual quarters can take different times to finish milking, the milking unit was not removed until it was observed that all quarters exhibited a near complete absence of milk flow through the milking cluster bowl.

Formalin-fixed *Staph. aureus* and saline intramammary infusions were prepared and aseptically infused as detailed previously (Gammariello et al., 2024). Infusate volumes were 20 mL/quarter.

Milk SCC were transformed into SCS like in Norman et al. (2000) to satisfy assumptions of equal variance; SCC <12,500 cells/mL were increased to this minimum value because lesser counts cannot be transformed. Energy-corrected MY (**ECMY**) were computed as described by Western et al. (2020) and total milk component yields were computed as in Gammariello et al. (2024). Milk components, MY, and ECMY were analyzed in separate models using PROC MIXED (SAS 9.4, SAS Institute Inc., Cary, NC). Fixed effects of the models were udder half treatment (n = 2), time point (n = 29), and their interaction; measures were repeated on udder halves interacting with cows over time. Time point main effects were not scrutinized for reasons we detailed previously (Gammariello et al., 2024) because cow-level responses to *Staph. aureus* challenges are confounded with changes in extraneous management and experimental factors over time. Accordingly, it is not possible to ascribe responses over time to the effect of time since challenge. For such a comparison, control cows that had only been treated with saline would be required, but this was not the purpose of this study. Conversely, differences between treatments within time points are the focus of this study, and least squares means were computed and contrasted using Fisher's least significant difference test within time point.

The initial intramammary challenge did not induce clinical signs of disease, but cows did develop clinical signs of mastitis after repeated challenges. Specifically, milk from FX-STAPH halves often yielded flakes and FX-STAPH udder halves would often be red and slightly swollen after challenge; 1 cow developed a febrile response 12 h after the final challenge (zenith of 39.67°C) that resolved by 24 h postchallenge. The SCS of SAL and FX-STAPH udder halves were similar at the time of the first challenge (d 0, *P* = 0.28), but SCS of FX-STAPH udder halves increased, and were always greater than that of SAL, for all subsequent postchallenge milkings (*P* ≤ 0.02; Figure 1A). In response to the first challenge, SCS of FX-STAPH halves peaked 24 to 36 h postchallenge. Notably, the SCS response to subsequent challenges was visibly greater in magnitude and faster (peaking 12 h postchallenge) than that of the initial challenge (Figure 1A). The SCS of SAL udder half remained low, always being less than a SCS of 3 (equal to an SCC of 100,000 cells/mL) for all but a single instance during the study period. Total somatic cell yields are presented in Figure 2B; somatic cell yields followed a pattern like that of SCS, being similar between treatments at the first challenge (*P* = 0.33), and markedly greater in FX-STAPH udder halves at all subsequent milkings (*P* ≤ 0.03).

**Figure 1:**
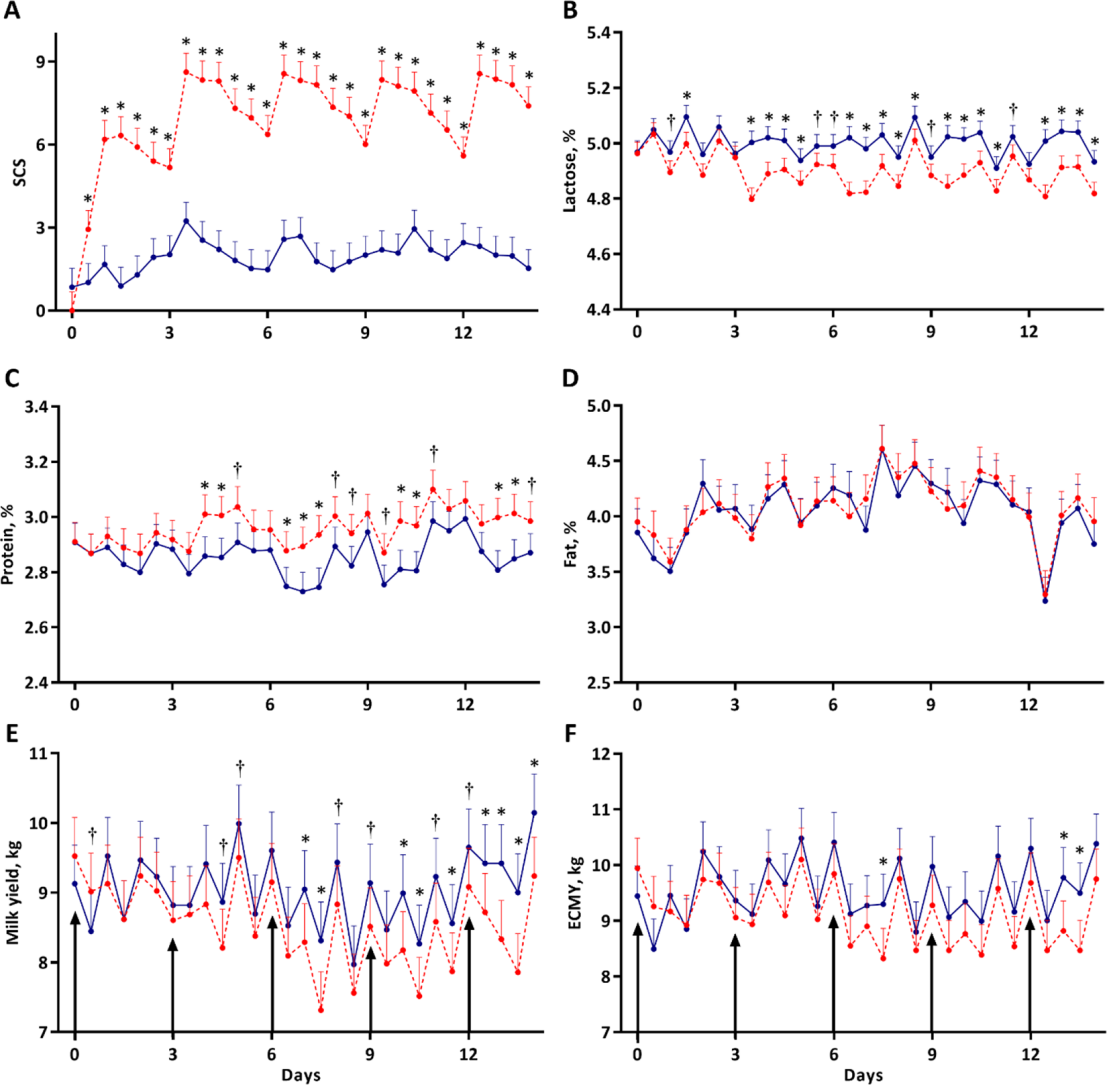
Mean milk SCS (A), lactose percentages (B), protein percentages (C), fat percentages (D), milk yields (E), and energy-corrected milk yields (ECMY, F) of udder halves that were repeatedly infused with saline (SAL, solid blue line, n = 4) or formalin-fixed *Staphylococcus aureus* (FX-STAPH, dashed red line, n = 4) on d 0, 3, 6, 9, and 12 after the first daily milking (signified by arrows). The milk yield and ECMY y-axes are truncated to best visualize the separation of udder half treatment means. Error bars denote the SEM. † Indicates means differ at *P* ≤ 0.10 within time point, and * indicates means differ at *P* ≤ 0.05 within time point.

**Figure 2:**
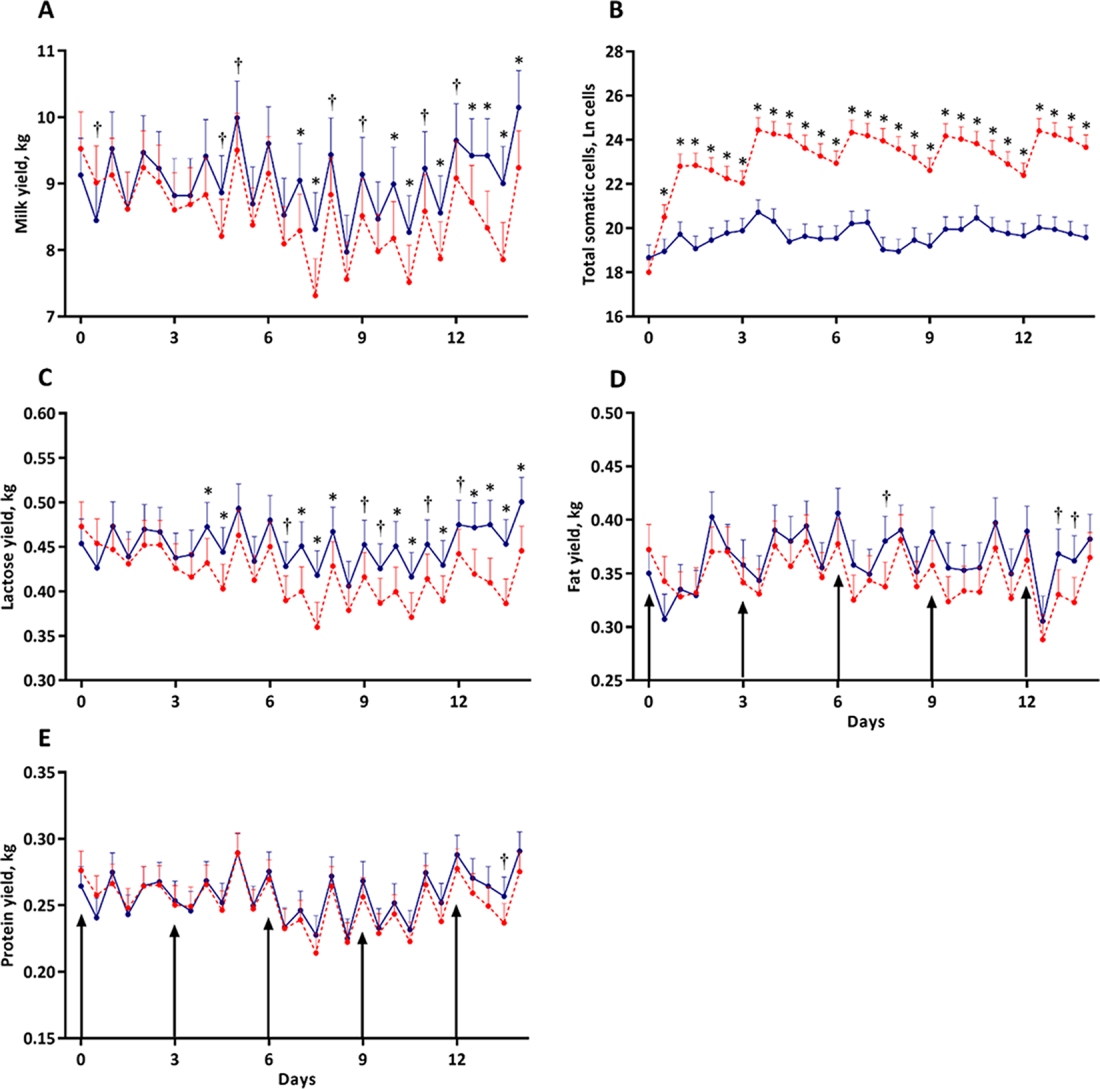
Mean milk yield (A), total somatic cells (B), and lactose (C), fat (D), and protein (E) yields of udder halves repeatedly infused with saline (SAL, solid blue line, n = 4) or formalin-fixed *Staphylococcus aureus* (FX-STAPH, dashed red line, n = 4) after the first daily milking on d 0, 3, 6, 9, and 12 (vertical arrows). Milk yields in panel A are the same as those in Figure 1E and are re-presented for comparative purposes to highlight the parallel relationship between milk yield and the total yield of lactose, fat, and protein. Error bars denote the SEM. † Indicates means differ at *P* ≤ 0.10 within time point, and * indicates means differ at *P* ≤ 0.05 within time point.

Milk lactose, protein, and fat percentages were comparable between SAL and FX-STAPH udder halves at first challenge (*P* ≥ 0.67; Figure 1B–D). Milk lactose percentages of FX-STAPH halves were temporally reduced after first challenge and then were either marginally (0.05 < *P* ≤ 0.1) or significantly (*P* ≤ 0.05) lower than the lactose percentages of SAL halves for all milkings after the second challenge (Figure 1B). Milk protein percentages behaved opposite of the lactose percentage pattern and were sometimes marginally (0.05 < *P* ≤ 0.1) or significantly (*P* ≤ 0.05) greater in FX-STAPH udder halves than SAL halves after the second challenge (Figure 1C). Milk fat percentages did not differ between FX-STAPH and SAL udder halves (*P* = 0.39; Figure 1D).

Although mean milk production clearly differed among time points (Figure 1E; *P* < 0.01), particularly resulting from a marked a.m./p.m. oscillation, SAL MY remained reasonably stable throughout the study. Conversely, FX-STAPH MY progressively decreased over time. Specifically, FX-STAPH halves began to exhibit reduced secretory capacity after the second challenge, 5 d into the trial (Figure 1E). When the entire study is considered, FX-STAPH udder halves yielded a mean of 0.49 ± 0.08 kg less milk than SAL udder halves at each milking (*P* < 0.01); when the MY of only the final FX-STAPH challenge were analyzed, MY losses averaged 0.96 ± 0.25 kg (*P* = 0.01) per milking. These outcomes indicate that the secretory capacity of FX-STAPH udder halves was increasingly negatively affected with each subsequent challenge.

Milk lactose, protein, and fat yields were comparable between SAL and FX-STAPH udder halves prechallenge (*P* ≥ 0.27; Figure 2C–E). Lactose yields were similar at all milkings after first challenge, but then intermittently became marginally (0.05 < *P* ≤ 0.1) or significantly (*P* ≤ 0.05) reduced after the second intramammary challenge (Figure 2C). Conversely, protein yields were similar between FX-STAPH and SAL halves throughout the trial except for a single time point after the final challenge when FX-STAPH halves yielded marginally less milk protein than SAL udder halves (*P* = 0.09; Figure 2E). Milk fat yields were only marginally lower in FX-STAPH udder halves at 3 time points throughout the study (0.05 < *P* ≤ 0.1; Figure 2D).

To our knowledge, this is the first development of a subclinical or mild clinical mastitis challenge model that elicits a MY disparity between challenged and unchallenged udder halves, where MY of the control udder is not apparently affected. Previous mastitis udder half studies employing potent immunostimulants such as LPS report marked MY reductions in the control udder halves, ranging from ∼15% (Shuster et al., 1991) to ∼60% (Shangraw et al., 2020). Notably though, in our prior attempts using a less potent immunostimulant, we were unable to elicit a meaningful MY response in the challenged udder half. The results of this study, when considered with our and others' prior works, indicate that duration of the inflammatory response during sterile subclinical and mild clinical mastitis is a chief determinant in dictating MY losses. Indeed, Plastridge (1958) summarized that *Streptococcus agalactiae* IMI progressively reduces MY over time, and that duration of infection affected the degree of fibrosis present in the affected quarter. Although mastitis pathogens vary in pathogenicity and virulence factors, our decision to use a sterile challenge agent isolates the milk loss and compositional alterations to the effects of the cow/host and removes contributions from a viable pathogen. This challenge model can be used to study the host-specific mechanism that contribute to MY loss and changes in milk composition at the level of the mammary gland.
